# Sleep and REM sleep disturbance in the pathophysiology of PTSD: the role of extinction memory

**DOI:** 10.1186/s13587-015-0018-9

**Published:** 2015-05-29

**Authors:** Edward F. Pace-Schott, Anne Germain, Mohammed R. Milad

**Affiliations:** Department of Psychiatry, Harvard Medical School, Massachusetts General Hospital—East, CNY 149 13th Street Room 2624, Charlestown, MA 02129 USA; Department of Psychiatry, University of Pittsburgh, Pittsburgh, PA USA

**Keywords:** Extinction, Sleep, REM sleep, PTSD, Anxiety, Insomnia, Stress

## Abstract

Post-traumatic stress disorder (PTSD) is accompanied by disturbed sleep and an impaired ability to learn and remember extinction of conditioned fear. Following a traumatic event, the full spectrum of PTSD symptoms typically requires several months to develop. During this time, sleep disturbances such as insomnia, nightmares, and fragmented rapid eye movement sleep predict later development of PTSD symptoms. Only a minority of individuals exposed to trauma go on to develop PTSD. We hypothesize that sleep disturbance resulting from an acute trauma, or predating the traumatic experience, may contribute to the etiology of PTSD. Because symptoms can worsen over time, we suggest that continued sleep disturbances can also maintain and exacerbate PTSD. Sleep disturbance may result in failure of extinction memory to persist and generalize, and we suggest that this constitutes one, non-exclusive mechanism by which poor sleep contributes to the development and perpetuation of PTSD. Also reviewed are neuroendocrine systems that show abnormalities in PTSD, and in which stress responses and sleep disturbance potentially produce synergistic effects that interfere with extinction learning and memory. Preliminary evidence that insomnia alone can disrupt sleep-dependent emotional processes including consolidation of extinction memory is also discussed. We suggest that optimizing sleep quality following trauma, and even strategically timing sleep to strengthen extinction memories therapeutically instantiated during exposure therapy, may allow sleep itself to be recruited in the treatment of PTSD and other trauma and stress-related disorders.

## Review

### Introduction

This review explores the possibility that disruption of sleep by acute or chronic stress may lead to alterations in emotional memory processing and, thereby, contribute to psychiatric illnesses such as post-traumatic stress disorder (PTSD) [1]. Here, one particular form of emotional memory, extinction of a conditioned fear response (i.e., learning that something that once signaled danger no longer does so) is emphasized. Extinction is a form of emotional memory that is important to normal emotion regulation [2], influenced by normal sleep and its disturbance [3–5], impaired in anxiety disorders [6], and exploited in their treatment [7]. Recent experimental findings, which are reviewed in reference [8], suggest that sleep may play key roles in the consolidation, integration, and balance of fear and extinction memory. The current review focuses on clinical issues and puts forward the hypothesis that one mechanism leading from psychological trauma to PTSD is stress-related sleep disturbances that interfere with sleep-dependent consolidation of emotion-regulatory neuroplasticity such as fear extinction and habituation.

### Disturbances of sleep and of emotions are reciprocally related

Healthy sleep is associated with normal emotion regulation [9, 10]. Conversely, sleep disturbance is both a common behavioral sequela of acute and chronic stress [11, 12] and a prominent symptom of anxiety and mood disorders [13, 14]. Specifically, sleep disturbance is a characteristic sequela of psychological trauma although subjective reports often indicate far greater severity than objective measurements in the immediately post-trauma period [15, 16]. While daytime affective symptoms and associated neural, physiological, and endocrine disturbances can adversely affect sleep, there is growing evidence that sleep disturbances (e.g., insomnia) can reciprocally impact daytime symptoms. For instance, epidemiological and prospective studies show that sleep disturbances that are present prior to trauma exposure, or that occur soon after trauma exposure, are a robust risk factor of poor psychiatric outcomes including PTSD, anxiety disorders, mood disorders, suicidality, and alcohol/substance use disorders [17–20]. Similarly, pre-existing insomnia has been shown to be a risk factor for incident depression [21–23]. The presence of untreated sleep disturbances comorbid with psychiatric disorders tends to attenuate treatment response and increase the risk of relapse [24–27]. Conversely, the persistence of consolidated sleep following stress or trauma exposure, as well as sleep improvements over the course of treatment for affective disorders, are associated with better mental health outcomes [28, 17].

As a result of such observations, it has been widely hypothesized that sleep disturbance is crucially involved in the etiology of PTSD rather than being solely a symptom arising secondarily from this disorder [16, 29–36]. In a comprehensive review on the temporal sequence of sleep disruption following traumatic events and the subsequent emergence of PTSD, Babson and Feldner [16] have shown that, in many cases following psychological trauma, subjective and, to a lesser extent, objective sleep disturbances can precede PTSD diagnosis thus providing clear evidence that such an etiological role of sleep is a distinct possibility. They note, however, the study of potential mechanisms for such a role is only in its infancy. The current review begins to explore evidence of one such factor, impaired fear extinction.

Involvement of sleep disturbance in the pathophysiology of PTSD does not, of course, exclude the more traditional view that psychiatric illness produces unique sleep disturbances or exacerbates pre-existing ones. Moreover, it is likely that a third vulnerability factor, such as individual variability in the degree to which psychological stress provokes enduring arousal in central limbic and autonomic circuits, can contribute to both poor sleep and increased risk of psychopathology. For example, waking hypervigilance and sleep disturbance could both arise from excess sympathetic activation without a direct interaction between the waking and sleep effects of such hyperarousal. As discussed below, chronic hyperarousal is increasingly implicated in the development of insomnia [37–42]. Similarly, repetitive nightmares and daytime traumatic memory intrusions may reflect a similar priming or disinhibition of retrieval for stored representations of the traumatic event, again without direct interaction between these two phenomena. Moreover, it has been suggested that sleep loss may secondarily diminish daytime coping strategies increasing the likelihood of developing psychopathology. Similarly, nightmares may sensitize individuals to waking trauma cues, or sleep disruption may directly exacerbate anxiety (reviewed in [16]). As in other disorders of biological systems, it is likely that pathogenic factors interact and that impaired negative feedback, escalating positive feedback, or compensatory allostatic mechanisms allow abnormalities in one domain to exacerbate those in others [43]. Therefore, we suggest that sleep disturbance and its negative effect on extinction memory is one of a number of neurocognitive and physiological pathways that could exacerbate risk of developing PTSD following a traumatic experience. For example, other neurocognitive factors potentially escalating risk of PTSD following initial trauma might include persistent threat of re-traumatization (enhanced conditioning), whereas physiological factors might include poor nutritional status (impairment of memory processing).

### The temporal development of PTSD following psychological trauma

Before proceeding to consider how sleep disturbance following trauma could contribute to the development of PTSD, it must first be established that PTSD is a disorder that can, in fact, develop over time following trauma rather than simply being an acute stress disorder (ASD) [1] that persists beyond an arbitrary 1-month threshold [1, 44]. What is the evidence that this is the case?

First, in a systematic review of prospective studies, among 19 studies of adults, following a median 6-month follow-up, a median of only 50 % of those with ASD subsequently met criteria for PTSD, whereas a median of only 47 % of those with PTSD previously met criteria for ASD [45]. Second, in a study of over 1000 traumatic injury survivors, only about a third of persons who developed PTSD by 1 year following a traumatic event showed ASD immediately following the trauma [46]. Importantly, this percentage increased by only about 9 % when a more liberal (subsyndromal) definition of ASD, not requiring dissociative symptoms, was used [46]. A similar percentage of persons with ASD (36 %) or subsyndromal ASD (30 %) went on to develop PTSD, although 65 % did eventually develop some psychiatric disorder.

Third, among post-combat military populations, it is not uncommon for diagnosed PTSD to emerge only after a delay of several months post-deployment [47]. For example, 88,235 Army soldiers were evaluated immediately upon returning from the Iraq war with a self-administered Post-Deployment Health Assessment that included specific screening questions for PTSD [48]. These same individuals were then re-evaluated with an assessment that contained the same PTSD screening questions at a median of 6 months after finishing the first evaluation [47]. In this re-assessment, report of psychological distress was markedly higher and reports of PTSD symptoms increased from 11.8 to 16.7 % in active-service Army and from 12.7 to 24.5 % in Reserve and National Guard [47]. Notably, among those who had reported PTSD symptoms on the immediately post-deployment assessment, approximately half reported improvement of these symptoms at re-assessment [47]. Therefore, the increased proportion of individuals reporting PTSD symptoms at re-assessment must have included individuals in whom symptoms appeared following their first assessment.

Fourth, although delayed-onset PTSD, defined most strictly as onset of any PTSD symptoms only after 6 months or more following trauma, is controversial and rare [49, 50], exacerbation of existing symptoms is common [49]. For example, a comprehensive review reported that, over the 6 months following a traumatic experience, worsening of existing PTSD symptoms or re-emergence of previously experienced symptoms was reported by 15.3 % of civilians and 38.2 % of military personnel [49]. Therefore, sleep disturbance may directly worsen existing symptoms, or, as here suggested, result in a failure to ameliorate such symptoms through consolidation of naturalistic or therapeutic extinction learning. In either case, the characteristic PTSD symptoms of intrusions (including nightmares), avoidance, negative affect, and hyperarousal [1] clearly may emerge or worsen over the initial months following a traumatic event.

### Fear conditioning and extinction

**Fig. 1:**
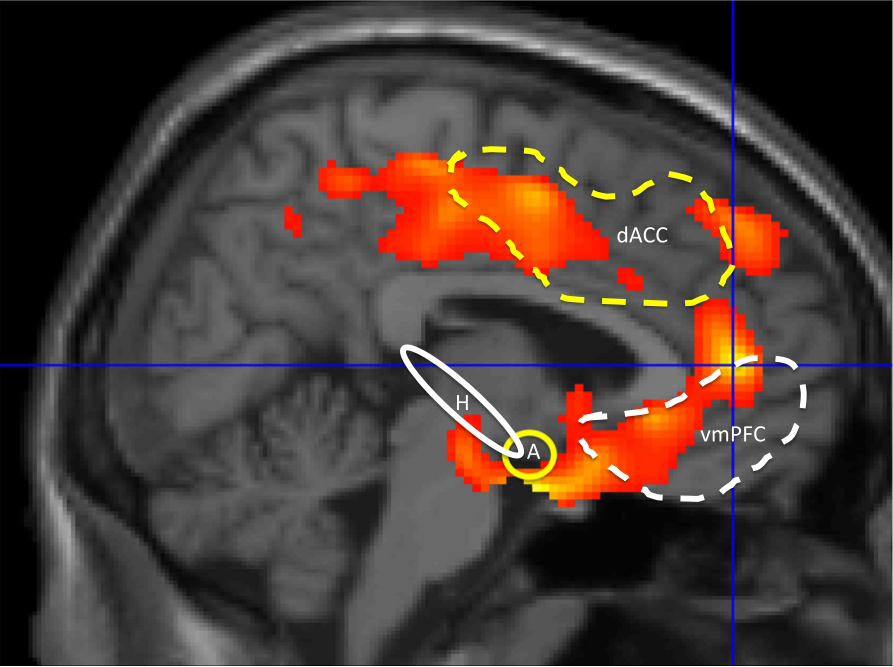
The “anterior paralimbic REM activation area” overlaps with fear and extinction circuits. ^18^Fluoro-deoxyglucose PET image of areas that reactivate during REM sleep following relative quiescence during NREM sleep. *Dashed lines* surround approximate cortical regions commonly activated in experimental protocols during fear conditioning (*yellow lines*) and during recall of extinguished conditioned fear (*white*) based upon Milad and Rauch [61], Fig. 3. *Solid lines* encircle approximate anatomic loci of subcortical structures similarly activated during fear conditioning (*yellow*) and extinction recall (*white*). The anterior paralimbic REM activation area includes the amygdala (*A*), and regions of dorsal anterior cingulate (*dACC*) and insular (not shown) cortices linked to a putative fear expression network. Additionally, this region includes the ventromedial prefrontal (*vmPFC*) and hippocampal (*H*) areas [127–129] linked to a putative extinction memory network

Fear conditioning occurs when an emotionally neutral stimulus is associated with an inherently aversive experience (unconditioned stimulus or US). The neutral stimulus thereby becomes a conditioned stimulus (CS) with the capability, on its own, to evoke a fearful conditioned response (CR). When the CS is subsequently presented repeatedly without the US, extinction (reduction) of the CR typically takes place. However, rather than erasing the CS-US association, extinction represents formation of a new memory, an “extinction memory,” signifying “CS-no US,” that competitively inhibits the memory of the CS-US contingency and expression of its associated CR when the CS is again encountered [51–59]. Neuroimaging research using de novo fear-conditioning and extinction paradigms has revealed areas in the brain associated with experiencing conditioned fear (a “fear expression network”) in the amygdala and dorsal anterior cingulate cortex (dACC) and other areas associated with memory for the extinction (inhibition) of this fear (an “extinction memory network”) that includes the hippocampus and ventromedial prefrontal cortex (vmPFC) [8, 59–61] (Fig. 1).

Extinction learning, viz. learning the “CS-no US” contingency, is the neurobehavioral basis for the efficacy of exposure therapy [7, 62]. Means by which memory for this therapeutic learning may be strengthened, and relapse to dominance of the fear (CS-US) memory prevented, are currently the subject of extensive clinical research [62–64]. It is also, however, important to recognize that extinction is a process that is ongoing in the course of everyday life. For example, individuals who display resilience and recovery, without any therapeutic intervention, following a psychologically traumatic event, presumably acquire extinction memories based upon spontaneous encounters with reminders of the trauma. And these extinction memories, in turn, prevent subsequent trauma cues from triggering fearful responses. And, as is the case for other forms of emotional memory [10], healthy sleep may be of ongoing, and cumulative importance in the consolidation of memory for both therapeutically induced and naturally learned extinction.

### Sleep-dependent memory consolidation

Extinction memory must be encoded, consolidated, and then retrieved in order to oppose conditioned fear. For declarative and procedural forms of memory, sleep has been widely demonstrated to promote the consolidation stage of memory formation [65–68], including processes related to prioritization and integration of newly acquired memories with existing stores [69–72]. Moreover, prior sleep can facilitate memory retrieval via such functions as protection from retroactive interference [73] and enhanced updating during reconsolidation [74]. Rapid eye movement (REM) sleep is associated with consolidation of emotional memory (reviewed in [8–10]), and REM sleep has been suggested to be the stage of sleep during which emotion is regulated [75]. For example, in the “Sleep to Remember, Sleep to Forget” model, Walker and colleagues suggest that REM sleep serves the dual purpose of consolidating the content of emotional memory and diminishing the memory’s emotional charge [75, 10]. Similarly, regulation of mood and working through of emotional responses to intra- and interpersonal stressors have been linked with REM sleep and associated dreaming [76–78]. Significantly, a broad anterior midline area of cortex and subcortex (the “anterior paramedian REM sleep activation area” detailed below) selectively activates during REM sleep following relative deactivation during non-REM (NREM) sleep [79], and this region encompasses both the fear expression and extinction memory networks (Fig. 1).

Physiological processes underlying sleep’s effects on memory consolidation have been demonstrated in animals and include replay, during sleep, of patterns of hippocampal place-cell firing that accompanied learning [80, 81]. Similar encoding-induced changes in subsequent sleep physiology are reported human polysomnographic and neuroimaging studies (reviewed in [67]). Post-learning sleep may facilitate synaptic, second messenger, gene transcription, and protein synthesis steps required for memory consolidation [82] such as *N*-methyl-D-aspartate (NMDA) receptor-dependent hippocampal long-term potentiation [83, 84]. Critical periods requiring sleep, including specifically REM sleep, for memory consolidation following encoding have been demonstrated in animals and humans [85], and such a period for extinction memory has recently been demonstrated for REM sleep [86].

### Extinction and disorders with abnormal levels of anxiety

Abnormal levels of anxiety seen in Diagnostic and Statistical Manual of Mental Disorders—5th ed. (DSM-5) Anxiety Disorders and Trauma- and Stressor-Related Disorders suggest a deficiency in emotion-regulatory mechanisms. A deficit in the ability to encode, consolidate, or retrieve extinction memory is believed to play a role in the development and perpetuation of such disorders [6, 59, 61, 87].

Having hypothesized deficient extinction in PTSD, at what point in the processes of fear acquisition, extinction learning, and extinction memory does this problem arise? Deficient memory for extinction has been shown to differentiate individuals with PTSD from trauma-exposed controls at both the behavioral and neural levels [88–90]. Notably, in these particular studies, acquisition of fear conditioning and extinction learning did not differ between these groups [88–90]. Other studies have specifically implicated a deficiency in PTSD to use contextual information to disambiguate danger versus safety [91]. Such findings would suggest that a deficiency in emotional memory systems might be of primary etiological importance in PTSD as might be expected given the above-cited abundant evidence for sleep effects on memory consolidation. Nonetheless, other studies do suggest greater de novo fear conditioning in PTSD [92] and deficient acquisition of extinction [93–95]. In addition, the degree of recall of de novo fear conditioning may predict later development of PTSD symptoms [96, 97]. Moreover, enhanced physiological reactivity to acoustic startle stimuli compared to controls is also commonly noted in this disorder [98–100]. Therefore, lesser capacity to acquire extinction, possibly due, in part, to enhanced ability to acquire a conditioned fear that is itself related to augmented autonomic and limbic reactivity may also play a role, particularly in the hyperarousal symptoms of PTSD. Interestingly, such hyperarousal also produces sleep disturbance that, in turn, may further disrupt sleep-dependent memory processes as detailed below.

The gold standard treatment for certain disorders with abnormal levels of anxiety involves formation of therapeutic extinction using exposure therapy [7, 62]. In such treatment, the patient is exposed to imaginal, pictorial, video, virtual reality, or in vivo representations of feared stimuli for a sufficient duration that anxiety is experienced and withstood and the patient thereby develops a new, inhibitory memory that opposes subsequent fear responses [7, 62, 101, 102]. Exposure is especially effective when fearful symptoms are associated with specific stimuli as in the case of PTSD [103], social anxiety disorder [104], obsessive-compulsive disorder [105], and specific phobia [106]. It is especially important to promote the generalization of extinction memories acquired during exposure sessions to prevent the return of fear outside of the safe, therapeutic context [3, 62, 64, 107–110].

An important distinction made during exposure therapy is between within-session learning by which extinction/habituation is initially acquired and between-session extinction/habituation, or the persistence of such learning across time from one exposure session to another [62]. (The combined term “extinction/habituation” is used because habituation is difficult to differentiate from extinction in clinical practice [111].) Note, however, that typically in exposure therapy, within-session extinction/habituation is continued following each session in the form of exposure homework (e.g., [103, 112]); therefore, the encoding and consolidation of extinction/habituation is, in reality, an iterative process. Between-session extinction/habituation corresponds to memory for what was learned within session and thus requires consolidation to persist over time. In the case of between-session extinction, this involves consolidation of an associative memory (e.g., of the CS-no US contingency) and, in the case of between-session habituation, the consolidation is of neural changes corresponding to a non-associative learning process [8]. Current animal research suggests that within-session and between-session extinction are dissociable processes [113], and studies of exposure therapy also show that the degree of within-session extinction does not predict the extent of the between-session extinction that, cumulatively, leads to clinical improvement [62]. Consequently, much research has gone into ways to strengthen this new learning via the timing, spacing, and gradation of intensity of exposures, manipulations of aspects of the environments or stimuli in which it is carried out, pharmacological interventions to potentiate the encoding and consolidation of inhibitory memory, and prevention of spontaneous recovery, renewal, or reinstatement of fear responses [62–64, 101, 114]. Sleep strategically timed so as to promote consolidation of extinction memory constitutes a potential new technique directed toward this same goal [3, 115]. Memory consolidation processes also provide opportunity for extinction/habituation to generalize, and sleep appears to augment this process as well [3, 107]. Specific clinical implications regarding the use of sleep as a means to enhance extinction/habituation are discussed in the “Sleep and exposure therapy” section below.

### Brain bases of deficient extinction in PTSD

PTSD patients show structural abnormalities in limbic regions associated with extinction recall including the perigenual anterior cingulate, amygdala, and hippocampus [6, 116, 117]. These are accompanied by greater functional activation of the fear expression network (amygdala and dACC) and lesser activation of the extinction network (hippocampus and vmPFC) during de novo fear-conditioning and extinction experiments [59, 60, 118–120]. Compared with trauma-exposed controls, those with PTSD show greater amygdala activation during extinction learning, and, during extinction recall, lesser activation of the vmPFC and hippocampus but greater activation of the dACC [88]. Therefore, in PTSD, there is both hyperactivation of the fear expression and hypoactivation of the extinction memory networks [59, 60]. However, not all neuroimaging studies show functional differences between PTSD and trauma-exposed controls in all of these loci or at the same anatomic coordinates within them. While a complete review of this diverse literature is beyond the scope of this article, excellent reviews can be found in [116, 119–126].

Importantly, the midline limbic and paralimbic areas that selectively activate during REM sleep (Fig. 1) encompass these same networks that show structural and functional abnormalities in PTSD. For example, this “anterior paralimbic REM sleep activation area” [79] includes the amygdala, and regions of anterior cingulate and insular cortex [127–129] that are linked to a putative fear expression network [61]. Similarly, this region includes the ventromedial prefrontal and hippocampal areas [127–129] linked to a putative extinction memory network [61]. As noted, these fear-related structures are hyperactive and extinction-related areas hypoactive in PTSD [88, 130].

### Sleep and the anxiety-related disorders

These common mechanisms in etiology, perpetuation, and treatment suggest that factors that strengthen or weaken extinction, such as good and poor sleep, respectively, may apply similarly across anxiety, traumatic stress, and obsessive-compulsive disorders. Sleep disruption is a DSM-5 [1] diagnostic criterion for generalized anxiety disorder and PTSD, is common in panic disorder [131, 132], and appears, more subtly, in obsessive-compulsive disorder [133]. Because both sleep and extinction appear to be degraded in PTSD, their interaction represents one putative mechanism contributing to the development and persistence of PTSD symptoms. And because treatment of PTSD with exposure-based therapies relies upon formation and strengthening of extinction memory, the memory-enhancing function of healthy sleep may play a role in recovery and disturbed sleep in treatment resistance.

### Sleep disruption in PTSD

Degradation of subjective and/or objective sleep quality is commonly reported in studies of individuals with PTSD [13, 16, 31, 134–136]. Sleep disruption and repetitive nightmares meet DSM-5 PTSD criteria for “alterations in arousal and reactivity” and “intrusion symptoms,” respectively [1]. For example, in a self-report study, the severity of PTSD symptoms predicted sleep problems to a much greater degree than age, gender, psychiatric comorbidity, type of trauma, or chronicity of PTSD [137]. Persistent trauma-related nightmares of a replicative nature are a near-universal symptom of PTSD [29, 138].

For objective sleep measures, a recent meta-analysis [134] found that, among the highly variable alterations of sleep in PTSD compared to control groups, increased stage 1 NREM sleep, decreased slow wave sleep (SWS) (see also [139]), and increased average number of rapid eye movements per minute in REM sleep (REM sleep density) were the most consistent abnormalities across studies. Additional abnormalities expressed by subgroups of PTSD patients included shorter total sleep time (TST), increased sleep onset latency, reduced stage 2 NREM sleep, and increased REM sleep as percent of TST [134, 140]. Polysomnographic studies have also shown that EEG spectral power at delta frequencies is significantly decreased in PTSD patients [139, 141, 142]. These abnormalities are consistent with an underlying hyperarousal in PTSD that lightens sleep, prevents deeper, more restorative sleep stages, and alters the distinct physiology of REM sleep [20, 29, 36, 134, 143]. Evidence that PTSD may influence the quality versus absolute quantity of REM includes not only greater REM density [134], but the fact that some studies have shown greater percent REM in PTSD [140, 139].

Therefore, both objective and subjective sleep disturbances represent core features of PTSD [36, 134, 136]. As noted, however, specific polysomnographic sleep abnormalities reported in different studies of PTSD are highly variable in type and severity [134] and can vary with age, sex, comorbidities, and other factors (for reviews, see [13, 134, 139, 144, 145]).

### Sleep abnormalities predict PTSD

**Fig. 2:**
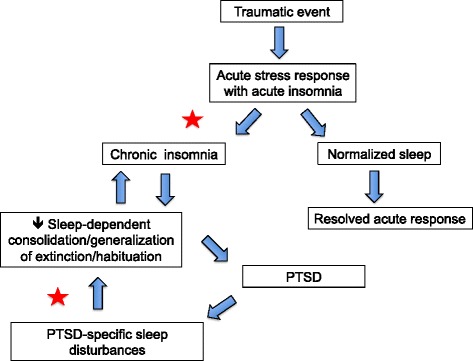
Possible pathway whereby sleep disruption accompanying acute response to trauma can lead to PTSD. In vulnerable individuals, acute post-traumatic insomnia can become chronic and disrupt processes of sleep-dependent emotional memory consolidation, thereby contributing to the etiology of PTSD. Chronic sleep disruption can subsequently perpetuate PTSD symptoms by continued interference with normal processing of emotional memories as well as impaired consolidation of therapeutic extinction memories if exposure therapy has been initiated. *Stars* indicate possible strategic points for sleep interventions to prevent PTSD onset or enhance exposure-based treatment

Objective and subjective sleep abnormalities, including insomnia complaints, that either precede or follow traumatic experiences, predict later development of PTSD (reviewed in [16, 33]). For example, motor vehicle accident survivors who later developed PTSD, unlike survivors who did not, had more severe immediately post-trauma sleep disturbances that failed to normalize over time [146]. Similarly, Mellman and colleagues showed that subjective insomnia, nightmare severity, and abnormalities of REM sleep, especially its fragmentation in the early aftermath of a traumatic injury, predicted later development of PTSD [20, 147–149]. In addition, higher sympathetic drive during REM sleep within 1 month of trauma was associated with development of PTSD symptoms at 2 months post-trauma [149]. Such sleep disruption might impede normal processing of emotional memories following trauma [20] including the ability to consolidate memory for the extinction of fear associated with traumatic memories (Fig. 2).

It is noteworthy that only a minority of individuals who have experienced a traumatic event develop PTSD. For example, among 99 studies on diverse disasters, the prevalence of PTSD at first assessment averaged 27 % [150]. Similarly, prevalence of PTSD in combat-exposed infantry is only around 20 % [151]. And, as noted above, only around 30 % of individuals with ASD go on to develop PTSD [46]. Therefore, factors other than trauma exposure alone or the acute reaction to trauma must contribute to the development of PTSD. In light of the preceding findings, we suggest that alterations in the emotion regulatory functions of sleep might be one such factor.

### Extinction and sleep in PTSD

Because sleep deprivation reduces amygdala-vmPFC functional connectivity [152] as well as task-related activation of the ventral anterior cingulate cortex (part of the vmPFC) in a positron emission tomography (PET) study [153], trauma-induced sleep loss might specifically impair consolidation of extinction memory via interference with vmPFC-amygdala circuitry. A finding that longer sleep on the night preceding functional magnetic resonance imaging (fMRI) scans was positively associated with both resting-state amygdala-vmPFC functional connectivity and higher self-report indices of mental health indicates that even mild restriction of sleep can diminish vmPFC-amygdala connectivity [154]. Animal studies show that stress-associated cues continue to interfere with REM sleep long after the original stressful experience [155–157], raising the possibility that REM sleep alterations in humans may play a role in both the acquisition and perpetuation of PTSD (Fig. 2). Although, there are not yet published studies on both sleep and extinction in PTSD patients, circuits involved in fear and extinction learning and memory are implicated in sleep-related symptoms of PTSD such as nightmares [138]. For example, in combat-exposed veterans with versus without PTSD, REM sleep is characterized by increased metabolic activity in the amygdala and anterior paralimbic regions, and reduced metabolism in hippocampal regions [158]. It is important to note that, in addition to effects of sleep on amygdala-vmPFC connectivity, trauma itself may affect such circuits, as is suggested by reports of structural abnormalities in these areas in PTSD [116, 120].

### Sleep-related neuroendocrine abnormalities in PTSD—relationship to emotional memory consolidation?

**Fig. 3:**
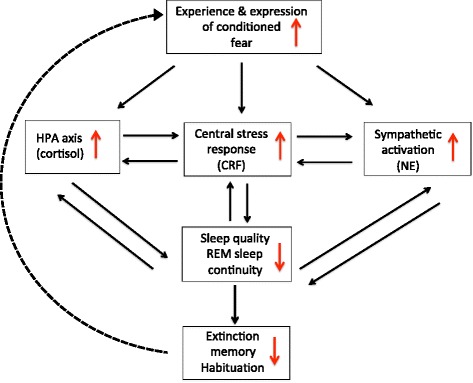
Hypothetical interactions among activated stress systems and disrupted sleep in PTSD. Note that multiple positive feedback loops result in depicted effects at any one node further driving effects at other nodes. Experimental evidence for many specific interactions depicted is provided in text. For clarity, the following mechanisms mentioned in the text are not depicted: 1) interaction between sympathetic activation and the HPA axis, 2) possible negative feedback mechanisms involving hypothalamic corticotropin releasing factor (CRF) that may explain hypocortisolemia in PTSD, and 3) direct effects of stress systems on extinction memory and habituation. The *dashed line* depicts an additional positive feedback mechanism whereby poor extinction memory promotes continued activation of neuroendocrine stress systems by failing to inhibit expression of conditioned fears. *HPA* hypothalamic-pituitary-adrenal, *CRF* corticotropin releasing factor, *NE* norepinephrine

By what mechanisms might both sleep and extinction memory become progressively degraded following traumatic stress? One possibility is that physiological stress responses produce sleep disruption that, via positive feedback, perpetuates these stress responses. In the rat, following experimental stress induction paradigms, sleep shows a number of compelling parallels to changes in human sleep following traumatic stress and in PTSD. For example, in the rat, fear conditioning and other forms of inescapable stress lead to disruption of sleep and fragmentation of REM sleep, conditioned reminders produce similar sleep-disruptive effects for several weeks post conditioning, and extinction training reverses these sleep effects (reviewed in ref. [157]). Such sleep disturbances in the rat have been linked with the actions of central stress systems including the sympathetic response, the hypothalamic-pituitary-adrenal (HPA) axis, and the central extra-hypothalamic stress system (reviewed in refs. [155, 157]). Within and between these stress systems, there are positive feedback mechanisms whereby neuroendocrine responses lead to elevated arousal and sleep disturbance that can, in turn, further activate stress responses. Abnormal activation of these stress systems has also been reported in PTSD, and these systems may interact following traumatic stress in a manner analogous to findings in animal models of stress. As depicted in Fig. 3, following a traumatic stressor, such interactions may disrupt sleep as well as sleep-mediated processing of extinction memory producing an escalating abnormality potentially leading to PTSD. The following section first describes neuroendocrine abnormalities in these three stress systems reported in PTSD. We then examine their potential impact on fear and extinction memory and interactions with sleep.

#### Noradrenergic abnormalities [“Sympathetic activation (NE)”]

PTSD is associated with elevated levels of central [159, 160] and urinary [161] norepinephrine, including measurements taken during sleep [161]. Normal NREM sleep is associated with a marked decrease in sympathetic and increase in parasympathetic drive [162–164]. Central secretion of norepinephrine (NE), the catecholamine responsible for the acute sympathetic stress response, acts to oppose REM sleep [165], and NE normally declines with sleep onset and deepening NREM sleep to reach its nadir in REM sleep [165]. Because nocturnal secretion of NE may remain relatively elevated in PTSD [159–161], this may be one factor serving to fragment REM sleep [29]. The success of the alpha-adrenergic antagonist prazosin in treating PTSD nightmares is strong evidence of NE involvement in the pathophysiology of this disorder [166, 167].

#### HPA-axis abnormalities [“HPA axis (cortisol)”]

Persons with PTSD frequently show abnormalities of the HPA axis [168]. The initiating factor of the HPA response is corticotropin-releasing factor (CRF), a polypeptide neurohormone whose secretion by the paraventricular nucleus (PVN) of the hypothalamus triggers release of adrenocorticotropic hormone (ACTH) from the anterior pituitary leading to the secretion of adrenal glucocorticoids [169]. Paradoxically, although CRF is elevated in the cerebrospinal fluid (CSF) of patients with PTSD [170–172], abnormally *low* baseline levels of plasma cortisol are typically observed in this disorder [173], possibly due to downregulation of pituitary CRF receptors resulting from elevated CRF [174, 175].

#### Central actions of CRF [“Central stress response (CRF)”]

Although CRF triggers release of cortisol via ACTH and PTSD may be characterized by low peripheral (plasma) levels of cortisol, variations in levels of central CRF and plasma cortisol are dissociable. This is illustrated, for example, by their circadian rhythms [164]. CRF sampled hourly in the cerebrospinal fluid of healthy volunteers showed an evening acrophase and morning nadir [176]—directly opposite to the pattern of plasma cortisol [177]. In addition to its effect on the HPA axis, CRF from the PVN as well as from the central nucleus of the amygdala (CeA) is a key neuromodulator activating the central extrahypothalamic stress system via CRF1 receptors in the CeA, basolateral nucleus of the amygdala (BLA), bed nucleus of the stria terminalis (BNST), and locus coerulus (LC) [178, 179]. Activation of CRF receptors in the BNST is associated with persistent (versus acute) threat responses in the rat [180, 181]. BNST activation also tracks sustained anxiety in humans [182], and sustained anxiety may better predict symptoms of PTSD than acute fear responses [183].

#### Positive feedback between central stress systems, disrupted sleep and REM sleep, and impaired extinction memory as a putative pathway for the escalation of post-traumatic psychopathology

Figure 3 suggests that activation of central stress systems not only mutually elevates activity among these systems themselves but also produces sleep disruption—itself a stressor that can further activate stress systems. Here, we present evidence for mutual activation between stress systems, followed by evidence for their reciprocal relationship with disrupted sleep and, finally, by suggestion of how impairment of extinction by poor sleep can further exacerbate stress responses.

CRF-ergic activation promotes secretion of NE by the LC [184, 185]. In turn, increased NE can stimulate the PVN resulting in further CRF release and activation of the HPA and central stress responses [184, 186]. Therefore, NE and CRF can reciprocally stimulate release of the other to escalate central stress responses [178] (Fig. 3).

Exogenous CRF disrupts sleep [187], endogenous CRF promotes waking [188], and sleep deprivation elevates endogenous CRF [189]. Recent studies in rodents suggest that the stress-induced reduction in REM sleep is attributable to the actions of CRF [190–192] as is the more general post-stress sleep fragmentation [193]. Chronically disturbed sleep can produce a persistent elevation of sympathetic activity and central NE [164, 194, 195]. Increased NE, in turn, activates other stress systems via its action on subcortical limbic structures such as the amygdala [178]. For example, based upon studies using a single-prolonged stress model of PTSD in the rat [196], a specific noradrenergic mechanism during sleep has been recently proposed to act on hippocampal-prefrontal systems and impair processing of traumatic memories in PTSD [197]. Therefore, sleep deprivation or restriction can generate a positive feedback cascade whereby central stress responses and sleep disruption mutually reinforce one another (Fig. 3). Thus, trauma exposure may precipitate a failure of sleep-dependent neuroendocrine processes that normally promote return to emotional homeostasis via nocturnal reductions in catecholamine levels, sympathetic drive, as well as HPA axis, and central CRF-ergic activity. Such changes may contribute to the development of PTSD in vulnerable individuals.

However, in addition to interactions taking place entirely within the interacting physiology of stress and sleep, failure to extinguish fear may also exacerbate stress and further drive the potentially pathogenic physiological interactions described above. Deprivation, curtailment, and fragmentation of sleep, and specifically REM sleep, can affect the processing of emotional memory including the consolidation and generalization of extinction (reviewed in [8]). Moreover, the direct effects of stress and stress hormones on memory are multifold (reviewed in [198]), and memory for extinction of fear conditioning may be especially susceptible to stress effects [199]. Therefore, the persistence of conditioned fear, in the face of its failed extinction (dashed line in Fig. 3), may continue to activate stress systems and further exacerbate positive feedback mechanisms that lead to further impairment of extinction and the persistence of pathological fear.

The exact ways in which REM sleep is altered in the period following a traumatic event, as well as after PTSD symptoms have developed, are not yet fully understood, and as noted above, a simple consistent quantitative change is not observed. Nonetheless, there is suggestive evidence in the fragmentation of REM following trauma [147, 148] or following inescapable stress in the rat [157] as well as in the increased REM density once PTSD has developed [134] that hyperarousal of limbic structures during REM may be one characteristic abnormality. The neurochemical changes in arousal systems observed in PTSD detailed above may underlie or contribute to such limbic hyperarousal during REM sleep, and recurrent REM sleep nightmares may be a subjective manifestation. The effects of limbic hyperarousal in REM sleep on consolidation of conditioned fear and its extinction may be to bias consolidation processes taking place via neuronal replay and other mechanisms during sleep (reviewed in reference [8]) toward the fear expression and away from the fear extinction networks described above. The underlying changes in REM sleep in PTSD thus remain areas in need of additional study.

### Insomnia, emotional dysregulation, and PTSD

The preceding discussion reviewed evidence that sleep disturbance is a cardinal symptom of PTSD that can appear prior to and predict PTSD symptoms. We reviewed evidence that stress responses and sleep disturbance can mutually exacerbate each other via neuroendocrine systems that also show abnormalities in PTSD, and that such abnormalities could potentially interfere with extinction learning and memory. Evidence that experimental sleep manipulations can influence fear conditioning and extinction is reviewed separately in [8]. However, to what extent can sleep disturbance predating or acutely following trauma itself initiate these pathogenic events? Examining psychopathological correlates and consequences of insomnia may begin to address this question.

The ubiquity of chronic insomnia both as a primary disorder and comorbid with psychiatric [200, 201] and non-psychiatric [202] conditions suggests that it reflects a trait vulnerability that can be triggered by a variety of stressors. Stressful events are a significant predictor of insomnia with odds of incident insomnia increasing in a dose-response manner for each such event [203]. The following section considers insomnia as a potential contributor to PTSD.

#### Emotional dysregulation and hyperarousal in insomnia

Insomnia is associated with dysregulation of emotions pertaining to sleep itself [204, 205]. However, a more general emotional dysregulation is a characteristic of many individuals with insomnia [206] that can be reflected in personality variables [207] such as a tendency to internalize conflict [208] as well as by the high comorbidity of insomnia with mood and anxiety disorders [22, 32, 200, 209]. Such findings have led to the suggestion that emotional reactivity is both a risk and perpetuating factor for the development of chronic insomnia [206, 210].

Contributing to this emotional dysregulation is the now well-replicated evidence for chronic hyperarousal in insomnia [37–39]. Such hyperarousal is manifested in both peripheral [37] and central [38] physiology as well as in pre- and post-morbid cognitive style [40, 41] and sensitivity of sleep quality to acute stress [42]. Acute insomnia is ubiquitous following a wide variety of stressors [211], and insomnia following traumatic events [146] including combat [33] is predictive of later development of PTSD [16].

#### REM sleep disruption in insomnia

As noted above, there is strong evidence that REM sleep is important in the emotion-regulatory function of sleep. For example, REM sleep fragmentation following a traumatic event is predictive of later development of PTSD [147, 148].

Although early polysomnographic studies of insomnia reported little change or small reductions in REM sleep compared to good sleepers [212], there is now increasing evidence for both percentage reductions [204] and fragmentation [213–215] of REM sleep in insomnia. Being the stage of sleep with the highest level of forebrain arousal [38], REM sleep may also be the most vulnerable stage to disruption by awakenings due to chronic physiological and cognitive arousal. This is because, in this activated behavioral state, the brain is closer to its threshold for awakening [213, 214].

#### Neuroimaging studies of Insomnia

Evidence is accumulating that functional abnormalities in emotional regulatory networks that overlap with the fear expression and fear extinction networks also occur in insomnia. Patients with insomnia showed higher levels of arousal (greater glucose metabolism) during NREM sleep compared with good sleepers, and objective and subjective increases in sleep disruption were positively associated with metabolic activity in the anterior cingulate cortex [38, 216]. Poor quality of sleep may negatively impact the ability of the vmPFC to consolidate and later express extinction memory. During resting-state fMRI, functional connectivity between the amygdala and other brain areas was reduced in persons with insomnia compared with healthy controls [217]. Specifically, amygdala connectivity with the insula, striatum, and thalamus was reduced, again suggesting dysfunction in emotion regulatory circuits.

**Fig. 4:**
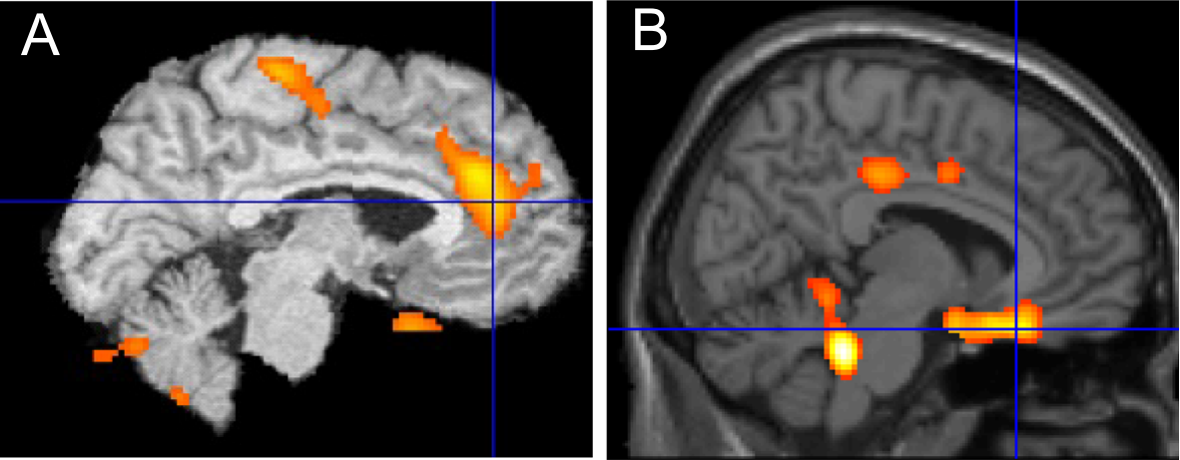
Comparison of REM activations in individuals with insomnia versus without insomnia. When comparing REM to wake, there is a greater increase in regional cerebral glucose metabolism (^18^fluoro-deoxyglucose PET) in an anterior midline region in close proximity to the region of the dACC that has been associated with fear expression (**a**). However, in a comparison of two different groups, the insomnia group showed lesser increase in the vmPFC, an area associated with the memory and expression of fear extinction (**b**)

Our studies suggest that insomnia patients show hyperactivation of the dACC and hypoactivation of the vmPFC during REM sleep. Using ^18^FDG-PET, insomnia patients showed a greater increase in cerebral glucose metabolism from wakefulness to REM sleep compared to good sleepers in an anterior midline region (Fig. 4a) in close proximity to the region of the dACC that has been associated with fear expression (Fig. 1). In addition, insomnia patients showed a smaller increase in cerebral glucose metabolism in the vmPFC from wakefulness to REM sleep (Fig. 4b). As noted, this latter area is associated with the memory and expression of fear extinction (Fig. 1). Thus, a closer investigation of the effects of chronic insomnia on fear learning and memory may provide novel insights into psychophysiological and neural mechanisms underlying anxiety and mood disorders.

#### Insomnia and PTSD

The normal, sleep-disrupting after-effects of a traumatic experience may develop into diagnosed chronic insomnia disorder^1^ or may be expressed as a more short-term, reactive sleep disturbance that does not meet the duration criteria of a chronic disorder.^2^ In either case, the likelihood that PTSD will later develop may increase due to the neurohormonal and mnemonic processes detailed above. Similarly, if an individual has poor sleep quality due to a pre-existing sleep disorder (such as obstructive sleep apnea) or is experiencing poor sleep due to limited sleep opportunity or sleep during an unfavorable circadian phase (as is common in the military), these same factors may increase vulnerability to PTSD irrespective of formal insomnia diagnoses. Indeed, among military service members, pre-deployment symptoms of insomnia have been shown to confer increased risk of post-deployment PTSD symptoms [17] and individuals with self-reported, pre-existing sleep problems had increased likelihood of developing PTSD following Hurricane Andrew [218]. Poor extinction memory may impair the ability to modulate arousal that results from stressors and thus could synergize with the physiological and cognitive hyperarousal of insomnia [37–40] to further elevate the risk of developing PTSD. Further evidence that insomnia can be primary is the fact that, whereas insomnia comorbid with anxiety disorders responds well to cognitive behavioral therapies developed for primary insomnia [209, 219], sleep disturbance often persists following successful treatment of PTSD [31]. Moreover, sleep-focused treatments can significantly improve both sleep and daytime symptoms of PTSD [220–222]. Therefore, insomnia may represent an emotionally dysregulated state that can contribute to the development of PTSD as well as exacerbate its symptoms and impede its treatment.

### Sex differences in extinction memory, insomnia and PTSD

Prevalence is greater in females than in males for both insomnia [223] and PTSD [224]. Translational studies with both humans [225–229] and rodents [230, 227, 228] have shown that extinction memory is sexually dimorphic (better in males) and that it varies across the menstrual cycle in females [225, 226]. Gender differences in the relationship between PTSD and sleep are also now being reported [144, 231]. For example, following a traumatic event, females who progressed to PTSD showed greater wake time after sleep onset than males who similarly developed PTSD [231]. Sex differences in the sleep symptoms of existing PTSD are also noted. For example, a study comparing sleep in PTSD and healthy controls in both sexes reported a gender × diagnosis interaction whereby, among females, those with PTSD showed greater REM duration and percent than controls whereas, among males, this difference appeared (non-significantly) in the opposite direction [139]. Additionally, as in the case of extinction memory in the experimental setting [232], it has been suggested that sleep symptoms in women may vary with hormonal levels and phase of the menstrual cycle [144].

### Sleep and exposure therapy

The ability to remember fear extinction is a key element of both normal recovery from trauma [118] and of psychotherapeutic treatment of PTSD using exposure therapy [7, 62, 101]. One mechanism by which sleep disturbance might precipitate or perpetuate PTSD is by preventing the consolidation and generalization of naturally occurring or therapeutically induced extinction memories during sleep [29]. The degree to which extinction learning can generalize from the specific stimuli extinguished in therapy to similar stimuli encountered outside the treatment setting will strongly impact the efficacy of such therapy [3, 62, 64, 108–110]. For example, fearful responding may re-emerge when the patient encounters an exemplar of a feared category of objects (e.g., spiders) that differs from the specific exemplar (e.g., species of spider) for which fear was extinguished in therapy [110, 233]. Similarly, gains achieved in exposure therapy may be compromised by fear renewal when the patient encounters a feared stimulus (e.g., a trauma reminder) outside of the therapeutic context in which it was extinguished [101, 108]. Such “return of fear” phenomena [234] may be conceptualized as re-emergence of conditioned fear due to failure of extinction memory to generalize from the treatment setting to diverse stimuli and settings that evoke such fears in the real world [63].

Extinction generalization may be particularly relevant to the treatment of PTSD, a disorder in which the opposing effect, generalization of fear responses, is ubiquitous [235]. Moreover, in PTSD, the same traumatic event can produce conditioned fear to multiple stimuli in multiple perceptual modalities each of which then becomes a warning signal of impending danger [236]. Generalization and multiplication of fear responses in PTSD may occur via processes such as second-order fear conditioning to primary trauma reminder cues [237]. Improved therapeutic extinction generalization thus might mitigate mechanisms by which fear generalization exacerbates the number and fear relevance of trauma reminders.

Clinical strategies to maximize extinction generalization include exposing patients to a variety of exemplars in a class of feared objects [101, 110], exposure of patients to feared stimuli in a variety of different contexts [101, 109] and in vivo exposure sessions [103]. A promising pharmacological approach for enhancing exposure therapy involves using D-cycloserine, an NMDA receptor partial agonist, that promotes NMDA-dependent memory consolidation of therapeutic extinction memory [238–240]. Some studies have suggested that outcomes from exposure therapy for PTSD can benefit from administration of D-cycloserine in temporal proximity to exposure sessions [241, 242]. Since, sleep [83] and, specifically, REM sleep [84] have also been shown to be important for NMDA-dependent long-term potentiation, sleep itself might be employed to help strengthen and generalize extinction [107].

In a preliminary application of this hypothesis to anxiety disorders [3], highly spider-fearful, young adult women were repeatedly exposed to a spider video after which half, who were exposed in the evening, had a normal night’s sleep and other half, exposed in the morning, had an equal (12-h) duration of continuous wakefulness. Following the delay, all groups viewed the same video and then videos of a new spider. Only in the wake group was there loss of psychophysiological and self-reported extinction and evidence of sensitization between sessions. Only the sleep group was there psychophysiological evidence of enhanced extinction retention and generalization between sessions. Because these effects did not differentiate control groups exposed and tested entirely in the morning or evening, a time-of-day explanation was ruled out. Thus, following exposure therapy, sleep may promote retention and generalization of extinction and prevent sensitization. These findings have been replicated in a recent study that used virtual-reality exposure therapy for DSM-IV diagnosed spider phobia [115]. Yet more recently, a large study of cognitive behavioral therapy in social anxiety disorder has shown that better self-reported baseline sleep was associated with better post-exposure treatment outcome on measures of anxiety [243].

### Important caveats

Impaired consolidation of extinction is unlikely to be the only sleep-related factor contributing to PTSD. Sleep disruption may lead to fatigue [244, 245], executive deficits [246, 247], mood dysregulation [10], and psychosocial impairments [248], all of which may degrade psychological resilience and exacerbate symptoms. Moreover, post-trauma disturbed sleep is unlikely, by itself, sufficient to produce PTSD—a disorder that also shows the above neuroendocrine abnormalities [161, 172, 173] as well as neurocognitive changes [249, 250], emergent psychosocial stressors [251], and genetic predispositions [252].

Caveats to animal models of physiological sleep disturbance and PTSD need also be emphasized. First, the sleep- and REM sleep-disruptive effects of experimental stressors appear with *inescapable* forms of stress, of which Pavlovian cued and contextual fear conditioning are canonical examples [157, 253] as, of course, are most traumatic events that precipitate PTSD in the human. In contrast, *escapable* shock, such as occurs in active avoidance learning paradigms, can instead lead to enhanced total and REM sleep with robust rebound of any loss resulting from the stress manipulation [156, 157, 253]. Therefore, aspects of the stressor such as controllability, predictability, and even the specific form of stress (e.g., restraint versus footshock) may produce different and even opposing effects on sleep and sleep-dependent mnemonic processes [156, 157]

Therefore, although putative pathways from traumatic stress to sleep disturbance and thence to poor extinction memory are compelling, the current state of knowledge cannot attribute development of PTSD solely or in part to disturbed sleep-dependent extinction, nor, indeed, to disturbed sleep alone. Nonetheless, among sleep-mediated sources of waking symptoms in PTSD, impaired consolidation and/or generalization of extinction memory during sleep remains a hypothetical mechanism highly suited to future investigation.

### Essential directions for future research

Despite the growing number of studies investigating sleep in PTSD, sleep and extinction memory, as well as extinction memory in PTSD and in anxiety disorders, there have been, to date, no studies specifically addressing sleep and extinction memory in patients with PTSD. Such studies, therefore, will be essential to test whether sleep-mediated effects on extinction memory seen in healthy subjects are altered in PTSD. Similarly, the interactions of extinction learning and memory with time-of-day [254] as well as sleep quality and chronotype [255, 256] described in healthy subjects (reviewed in ref. [8]) should also be examined in PTSD. Especially informative would be prospective longitudinal studies initiated following a traumatic event to monitor sleep physiology, circadian patterns of sleep-waking, HPA-axis function, mood, and nightmare frequency/content in order to examine potential linkages between these measures and emergent symptomatology in those individuals who progress to PTSD compared to those who prove resilient.

There have been some early attempts to examine sleep augmentation of pharmacological interventions that may be used to enhance exposure therapy. For example, in healthy volunteers following conditioning and extinction learning, valproic acid, a histone deacetylase inhibitor, enhanced delayed resistance to reinstatement following sleep but D-cycloserine enhanced such resistance following an awake delay [257]. Therefore, combining pharmacotherapy with strategically timed post-exposure sleep may further enhance exposure therapy [258].

Finally, future studies might also examine the effects of sleep on the newly described phenomenon of fear erasure using reconsolidation blockade following retrieval of traumatic memory [2, 57, 259–261]. Given findings that bidirectional plasticity, that includes depotentiation as well as long-term potentiation (LTP), may require REM sleep [197], it is possible that sleep following such reactivation would allow depotentiation to better compete with reconsolidation processes that require LTP. As in the pursuit of enhanced extinction, a sleep component might be added to pharmacological interventions designed to impede reconsolidation of aversive memory such as blockade of noradrenergic transmission [262, 263].

## Conclusions

Sleep, acting as a modulator of physiological stress and emotional memory, is of crucial importance in maintaining day-to-day emotional homeostasis and long-term mental health. Sleep disturbance predating or acutely resulting from a traumatic event, particularly if it develops into chronic insomnia, may initiate positive feedback and allostatic mechanisms that impair emotional regulation and promote the pathophysiology of PTSD. The findings reviewed herein have compelling research and clinical implications. First, the effects of sleep deprivation and restriction on extinction learning and recall as well as their neural bases in healthy individuals (reviewed in ref. [8]) should be further investigated. Second, the interaction of sleep deficit, extinction recall, and clinical diagnosis will require studies in which extinction learning and recall are visualized in the brains of PTSD patients with greater and lesser sleep disturbance and these findings compared to trauma-exposed controls as well as patients with non-PTSD-related insomnia. Importantly, however, clinical applications of accruing knowledge need not await definitive findings but can be concurrently investigated to help address the urgent need for innovation in treatments for PTSD. For example, just as disrupted sleep may impair emotional recovery during the critical period following traumatic stress, healthy sleep may be protective at this same point in time. As has often been suggested, proactive treatment of any acute sleep disruption may be a crucial first intervention in the prevention or early treatment of PTSD symptoms [20, 29, 31, 36]. Although the evidence here reviewed indicates the specific importance of REM sleep, behavioral techniques to selectively enhance this sleep stage (e.g., prior REM sleep deprivation) involve further sleep disruption. Therefore, to preserve REM sleep following a trauma, optimizing overall sleep quality by treating comorbid insomnia or other sleep disorders and improving sleep hygiene is important. Another consideration that requires more research is whether REM-sleep suppressive agents, such as many aminergic antidepressants, should be avoided in the early aftermath of trauma. There is also preliminary evidence that the alpha-1 adrenoreceptor antagonist, prazosin, which is effective in relieving nightmares in PTSD, may also serve to normalize REM sleep [264]. Additionally, evaluation of pre-existing sleep disorders may serve as a screening criterion to identify individuals entering high-stress professions such as the military or first responders who are at greatest risk of developing PTSD [17, 265]. Lastly, the memory-enhancing function of sleep might be exploited to strengthen therapeutic extinction learned in exposure-based therapy using strategically timed sleep bouts [3, 115].

## Abbreviations

1: BLA: basolateral nuclei (amygdala)
BNST: bed nucleus of the stria terminalis
CeA: central nucleus (amygdala)
CRF: corticotropin-releasing factor
CS: conditioned stimulus
CSF: cerebrospinal fluid
dACC: dorsal anterior cingulate cortex
DSM-5: Diagnostic and Statistical Manual of Mental Disorders—5th ed.
EEG: electroencephalography
fMRI: functional magnetic resonance imaging
HPA axis: hypothalamic-pituitary-adrenal axis
LC: locus coeruleus
LTP: long-term potentiation
NE: norepinephrine
NMDA: *N*-methyl-D-aspartate receptor
NREM: non-REM sleep
PSQI: Pittsburgh Sleep Quality Index
PTSD: post-traumatic stress disorder
PVN: paraventricular nucleus (hypothalamus)
REM: rapid eye movement
TST: total sleep time
US: unconditioned stimulus
vmPFC: ventromedial prefrontal cortex

## Footnotes

1 In various studies, diagnosed insomnia is typically DSM-IV [266] Primary Insomnia, DSM-5 [44] Insomnia Disorder, International Classification of Sleep Disorders Second Edition (ICSD-2) [267] Psychophysiological Insomnia, or ICSD-3 Chronic Insomnia Disorder [268] all of which have similar criteria including difficulty initiating or maintaining sleep, daytime fatigue, malaise or dissatisfaction with sleep, duration of at least 3 months (1 month in DSM-IV) and symptom occurrence on 3 or more days per week. Insomnia is frequently comorbid with mood or anxiety disorders in which case it is variously classified as Insomnia Due to Mental Disorder (ICSD-2, ICSD-3) Insomnia Related to another Mental Disorder (DSM-IV) or Insomnia Disorder with Non Sleep-Disorder Mental Comorbidity (DSM-5).

2 When sleep disturbance does not meet the duration criteria for the above definitions, it is variously classified as Situational/acute Insomnia (DSM-5), Adjustment Insomnia (ICSD-2), or Short-Term Insomnia Disorder (ICSD-3).
